# Novel Compound Heterozygous Variants in *TGM1* and *CYP4F22* in Two Newborns with Non-Syndromic Epidermal Differentiation Disorders (*TGM1*-nEDD and *CYP4F22*-nEDD)

**DOI:** 10.3390/jcm15145556

**Published:** 2026-07-15

**Authors:** Gregorio Serra, Giovanni Barbera, Vincenzo Antona, Danilo Malizia, Iria Neri, Enrico Perre, Maria Piccione, Annalisa Vetro, Alessandra Vancini, Mario Giuffrè, Giovanni Corsello

**Affiliations:** 1 Neonatal Intensive Care Unit, Azienda Ospedaliera Universitaria Policlinico “Paolo Giaccone”, Department of Health Promotion, Mother and Child Care, Internal Medicine and Medical Specialties “G. D’Alessandro”, University of Palermo, 90127 Palermo, Italy; g.barbera98@gmail.com (G.B.); vincenzo.antona@policlinico.pa.it (V.A.); d.malizia@villasofia.it (D.M.); mario.giuffre@unipa.it (M.G.); giovanni.corsello@unipa.it (G.C.); 2 Dermatology Unit, Sant’Orsola-Malpighi Hospital, IRCCS Azienda Ospedaliero-Universitaria di Bologna, University of Bologna, 40138 Bologna, Italy; iria.neri@unibo.it; 3 Neonatal Intensive Care Unit, Maggiore Hospital “Carlo Alberto Pizzardi”, AUSL Bologna, 40133 Bologna, Italy; enricoperre@gmail.com (E.P.); alessandra.vancini@ausl.bologna.it (A.V.); 4 Genetics Laboratory, Medical Genetics Operative Unit, Villa Sofia-Cervello Joint Hospitals, University of Palermo, 90146 Palermo, Italy; maria.piccione@unipa.it (M.P.); a.vetro@villasofia.it (A.V.)

**Keywords:** lamellar ichthyosis, self-improving collodion ichthyosis, collodion baby, *TGM1*, *CYP4F22*, novel mutation, targeted NGS

## Abstract

**Background**: Congenital ichthyoses are a clinically and genetically heterogeneous group of Mendelian disorders of cornification. According to the 2025 classification, non-syndromic epidermal differentiation disorders (nEDDs) are categorized based on the biological function of the causative gene products. Variants in *TGM1* cause a transglutaminase-related nEDD (*TGM1*-nEDD), typically corresponding to the traditional phenotype of lamellar ichthyosis (LI), whereas biallelic variants in *CYP4F22* cause a lipid-metabolism-related nEDD (*CYP4F22*-nEDD), formerly classified as ARCI type 5. The latter form is characterized by impaired acylceramide synthesis and epidermal barrier dysfunction. Although *CYP4F22*-nEDD usually follows a persistent course, considerable phenotypic variability has been reported, including milder forms and self-improving collodion ichthyosis. **Methods and Results:** We report two unrelated Italian neonates with congenital ichthyosis, investigated by targeted next-generation sequencing (NGS). Patient 1 presented with a phenotype consistent with LI and carried compound heterozygous *TGM1* variants: the known pathogenic c.1147G>A p.(Val383Met) variant, and the novel c.860C>T p.(Thr287Ile) variant, classified as likely pathogenic according to the American College of Medical Genetics and Genomics (ACMG) criteria. Patient 2 presented as a collodion baby and carried two heterozygous *CYP4F22* variants: the known pathogenic nonsense variant c.1084C>T p.(Arg362Ter), and the previously unreported in-frame deletion c.543_545del p.(Ile181del), currently classified as a variant of uncertain significance (VUS). Because parental segregation analysis was unavailable, the allelic phase of the *CYP4F22* variants could not be established, and molecular confirmation of autosomal recessive inheritance was not possible. Both patients showed marked clinical improvement during follow-up with topical emollient therapy alone. In Patient 2, the clinical course was consistent with a self-improving collodion ichthyosis phenotype. A structured literature review was performed to contextualize the clinical and molecular findings. **Conclusions**: These cases illustrate the clinical and genetic heterogeneity of nEDDs, and confirm the value of NGS in the management of neonates with congenital ichthyosis. The principal novelty of this report lies in the identification of previously unreported *TGM1* and *CYP4F22* sequence variants. While functional validation is lacking, integration of clinical and genetic findings with literature evidence may assist interpretation of rare variants and support genetic counselling.

## 1. Introduction

Congenital ichthyoses (CIs) represent a clinically and etiologically heterogeneous group of Mendelian Disorders of Cornification (MEDOC), typically involving most or all layers of the integument. The hallmark clinical features include generalized xerosis, scaling, and hyperkeratosis, often associated with erythroderma. The overall incidence is estimated at 6.7 per 100,000 live births. Classification is primarily clinical and distinguishes between non-syndromic forms—when ichthyosis occurs as an isolated skin disorder without extracutaneous features—and syndromic forms, in which the phenotype also involves other organs. Major non-syndromic forms include ichthyosis vulgaris (IV), X-linked recessive ichthyosis (XRI), keratinopathic ichthyoses (KPIs), and autosomal recessive congenital ichthyoses (ARCIs). According to the updated 2025 classification of Mendelian disorders of cornification, non-syndromic epidermal differentiation disorders (nEDDs) are further grouped according to the biological function of the causative proteins. Within this framework, *TGM1*-related disease is classified as transglutaminase-associated nEDD (*TGM1*-nEDD), whereas *CYP4F22*-related disease belongs to lipid-metabolism-associated nEDD (*CYP4F22*-nEDD). Traditional clinical designations such as harlequin ichthyosis (HI), lamellar ichthyosis (LI), congenital ichthyosiform erythroderma (CIE), and self-improving collodion ichthyosis (SICI) remain useful descriptive phenotypes. Keratinopathic ichthyoses constitute a distinct group of keratin-associated nEDDs, and are predominantly inherited in an autosomal dominant manner [1,2,3,4,5].

Lamellar ichthyosis (LI) presents with a milder phenotype than HI, although the severity of cutaneous manifestations varies widely among individuals. Characteristic features include large, thick, dark gray to brown scales covering most of the body surface. This broad phenotypic variability reflects the underlying genetic heterogeneity of the disease. Genes most frequently implicated in LI include ATP Binding Cassette Subfamily A Member 12 (*ABCA12*), Lipoxygenase-3 (*ALOXE3*), 12R-Lipoxygenase (*ALOX12B*), Ceramide Synthase 3 (*CERS3*), Cytochrome P450 Family 4 Subfamily F Member 22 (*CYP4F22*), NIPA-like Domain-Containing 4 (*NIPAL4*/*ICHTHYIN*), Patatin-like Phospholipase Domain-Containing Protein 1 (*PNPLA1*), and Transglutaminase 1 (*TGM1*) [3]. Mutations in *TGM1* represent the leading cause of ARCI and account for approximately 90% of LI cases. Biallelic variants in *CYP4F22*, which encodes a cytochrome P450 enzyme involved in epidermal lipid metabolism, are associated with *CYP4F22*-nEDD (formerly ARCI type 5), a subtype characterized by impaired ω-hydroxylation of ultra-long-chain fatty acids and defective acylceramide synthesis leading to skin barrier dysfunction; clinically, although patients typically develop a persistent ichthyosis phenotype, some degree of phenotypic variability has been reported, and evolution toward a milder presentation, including self-improving collodion ichthyosis, may be observed, although it appears to be uncommon. In recent years, diagnostic strategies for congenital ichthyosis have progressively evolved from sequential single-gene testing toward next-generation sequencing (NGS)-based approaches. Depending on local availability and clinical context, targeted gene panels, clinical exome sequencing or whole-exome sequencing are increasingly used as first-line diagnostic tools because of the marked genetic heterogeneity of these disorders. Targeted panels remain valuable when a well-defined group of candidate genes is suspected, whereas exome-based/whole genome approaches may improve diagnostic yield in atypical or genetically unresolved cases. The introduction of targeted next-generation sequencing (NGS) has markedly increased diagnostic yield, enabling the identification of pathogenic variants in about 80–90% of CI cases [6].

## 2. Methods and Results

Here, we describe two unrelated Italian newborns presenting clinical signs consistent with CI. NGS analysis identified compound heterozygous variants in *TGM1* in the first patient and in *CYP4F22* in the second one, providing molecular findings consistent with *TGM1*-nEDD in Patient 1 and suggestive of *CYP4F22*-nEDD in Patient 2. In order to strengthen the genetic characterization and to better support the possible genotype–phenotype correlation, we also included detailed bioinformatic analyses on the predicted protein function impact of the identified variants.

### 2.1. Patient 1

**Figure 1:**
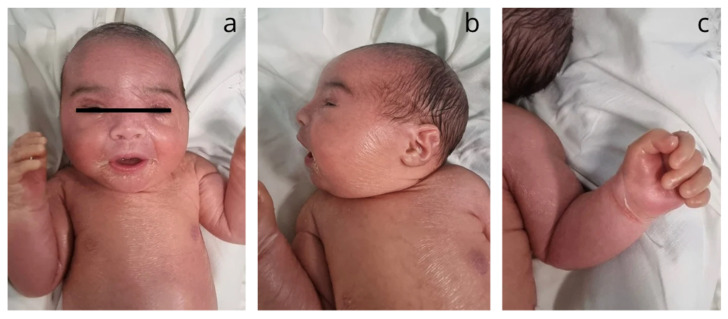
Patient 1 at birth: parchment-like skin, tight, shiny film enveloping the entire erythematous body surface, accompanied by some cracked and macerated areas. (**a**) Eclabium and ectropion. (**b**) Constriction of the auricular cartilages (“folded ears”). (**c**) Corneal thickening leading to semi-flexed position of fingers.

The patient was a female newborn delivered at 38 + 6 weeks of gestation by spontaneous vaginal birth. She was the second child of healthy, non-consanguineous parents. Family history revealed a brother affected by autism spectrum disorder associated with a *CDH8* gene variant. Targeted sequencing of *CDH8*, performed during the current pregnancy by chorionic villus sampling, yielded negative results. No additional genetic conditions or genodermatoses were reported in the family. Pregnancy was uneventful, and maternal screening for TORCH infections, as well as vaginal and rectal swabs for Streptococcus agalactiae (Group B Streptococcus), were negative. Serial ultrasound examinations demonstrated appropriate fetal growth and development. Delivery took place at a first-level birth center in Palermo, Italy. The neonate demonstrated good adaptation to extrauterine life, with Apgar scores of 9 and 10 at 1 and 5 min, respectively. Anthropometric parameters at birth were weight 3345 g (71st percentile), length 49 cm (50th percentile), and head circumference 34 cm (59th percentile), according to Italian INeS growth charts [7]. Immediately after birth, significant skin abnormalities were noted, raising suspicion of collodion baby syndrome. The patient was promptly transferred to the Neonatal Intensive Care Unit (NICU) at the University Hospital of the town. On admission, physical examination revealed parchment-like, shiny, tight skin covering the entire erythematous body surface, with fissures and macerated areas. Eversion of the lips (eclabium) and eyelids (ectropion) due to marked *stratum corneum* thickening were observed, together with joint stiffness. Auricular cartilage deformities (“folded ears”) and semi-flexed fingers related to corneal thickening were also present (Figure 1a–c).

Neurological evaluation showed weak sucking and mildly reduced palmoplantar grasp reflexes, while no abnormalities were detected in other organ systems. Routine metabolic screening, hematology, liver and kidney function tests, and markers of cytolysis were all within normal limits. Cranial, cardiac, and abdominal ultrasound examinations excluded associated congenital malformations. Ophthalmologic and audiologic assessments were unremarkable. Given the suspicion of congenital ichthyosis, targeted next-generation sequencing (NGS) was performed using a 54-gene panel associated with congenital skin disorders (Table 1). This revealed compound heterozygous variants in the *TGM1* gene: c.1147G>A p.(Val383Met) and c.860C>T p.(Thr287Ile). Segregation analysis confirmed inheritance from the mother and father, respectively. Both parents were, in fact, heterozygous carriers, while the older brother carried neither variant.

**Figure 2:**
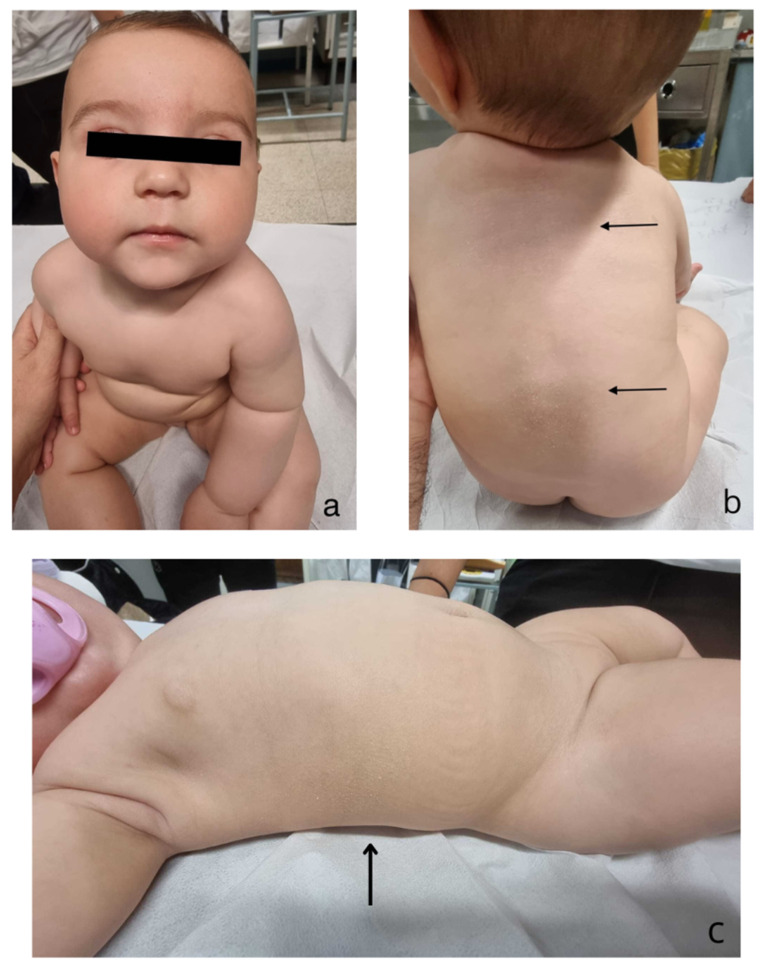
Patient 1 at age 7 months: (**a**) marked improvement of clinical manifestations on the face. Dry skin with large, fine, and grayish/brownish scales on (**b**) the posterior thoracic and lumbar regions, and (**c**) from the right axillary to inguinal area, more evident on the flank (indicated by arrows).

The neonate was placed in an incubator with 70% humidity to prevent hypernatremic dehydration and hypothermia. Parenteral nutrition was administered for the first 48 h to maintain electrolyte balance and ensure adequate caloric intake. Subsequently, enteral feeding with formula milk, as per maternal preference, was well tolerated. Dermatology and plastic surgery teams guided treatment, which included daily topical white petrolatum, fusidic acid cream twice daily on fissured areas, and cleansing with a vitamin-enriched, lipid-replenishing oil. This regimen was discontinued after two weeks due to erythema on the trunk, neck, and face, and was replaced by emollient cream alone. The patient was discharged after three weeks in good condition, with satisfactory weight gain and improved skin integrity. At discharge, only scaling on the back and limbs persisted. The patient was enrolled in a multidisciplinary program including dermatology, neurodevelopmental, and genetic follow-up. At 7 months of age, a significant clinical improvement was observed, with residual dysepithelialized areas on the back, right flank, groin, and axilla (Figure 2a–c).

**Figure 3:**
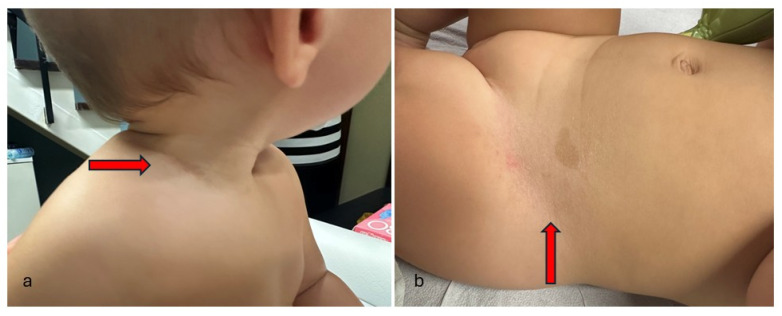
Patient 1 at age 9 months: dry skin with persistent mild lamellar desquamation on (**a**) the right latero-cervical region, and (**b**) left flank and inguinal areas (indicated by arrows).

At the most recent evaluation (9 months and 21 days), growth was appropriate (weight 9870 g, 90th percentile; length 70 cm, 33rd percentile; head circumference 44 cm, 47th percentile, according to WHO standards [8]). Examination showed only mild xerosis and lamellar desquamation on the right cervical region and left flank/groin (Figure 3a,b). Neuromotor development was normal, and no systemic abnormalities were identified.

#### 2.1.1. Genetic Analysis

DNA from the proband, her parents, and her older brother was analyzed using next-generation sequencing (NGS) with a paired-end protocol on a NextSeq 550Dx sequencer (Illumina, San Diego, CA, USA). Prior to sequencing, selective enrichment of coding regions of 54 genes associated with congenital ichthyosis (CI) was performed (Diatech-Twist Clinical Exome 2.0 kit; Diatech Pharmacogenomics, Jesi, Italy). Data processing was carried out with the DRAGEN Germline Pipeline and Variant Interpreter on the BaseSpace™ Sequence Hub (Illumina, San Diego, CA, USA), including read alignment to the reference genome (GRCh38/hg38) [9], as well as variant annotation and filtering.

Quality control thresholds were as follows: mean coverage ≥ 60 reads per nucleotide, >95% of target bases covered at ≥20×, and >92% covered at ≥30×. Variant interpretation was restricted to non-synonymous exonic variants and splice-site variants within ±2 nucleotides of coding exons, with population frequency < 1%. Variants were filtered based on the clinical indication and expected inheritance pattern. Exome analysis of the parents and older brother was limited to variants identified in the proband. All variants were annotated according to Human Genome Variation Society (HGVS) nomenclature [10] and classified following the American College of Medical Genetics and Genomics (ACMG) guidelines [11].

For variant interpretation, multiple resources were consulted, including the scientific literature, DECIPHER [12], ClinVar [13], the Human Gene Mutation Database (HGMD) [14], the Leiden Open Variation Database (LOVD) [15], the Italian Troina Database [16], as well as disease- and gene-specific repositories. Allele frequency was assessed using the gnomAD v4.1 population database [17] and the laboratory’s internal dataset. The laboratory performing the analysis is ISO 9001:2015 certified and participates in external quality assurance programs (GenQA, Genomics Quality Assessment) [18].

The maternally inherited c.1147G>A (p.Val383Met) mutation is well documented in the literature as pathogenically associated with lamellar ichthyosis (see below). The paternally derived c.860C>T p.(Thr287Ile) variant has not been previously reported as deleterious in the literature. However, according to ACMG criteria (PM1, PM2, PP2, PP3, PP4) [11], the variant was classified as likely pathogenic. Nevertheless, the pathogenic role of p.(Thr287Ile) remains supported primarily by ACMG classification, segregation data, phenotype consistency, and computational evidence, as no functional validation is currently available.

#### 2.1.2. Bioinformatic Analysis on Protein Function of Identified Variants

For bioinformatic analysis, computational predictions were obtained from Ensembl release 115 [19], incorporating several in silico tools:.SIFT [20], PolyPhen-2 [21], Combined Annotation Dependent Depletion (CADD) [22], Rare Exome Variant Ensemble Learner (REVEL) [23], MetaLR [24], and MutationAssessor [25]. These tools were used as supportive evidence for variant interpretation. The results are summarized in the Supplementary File S1.

### 2.2. Patient 2

**Figure 4:**
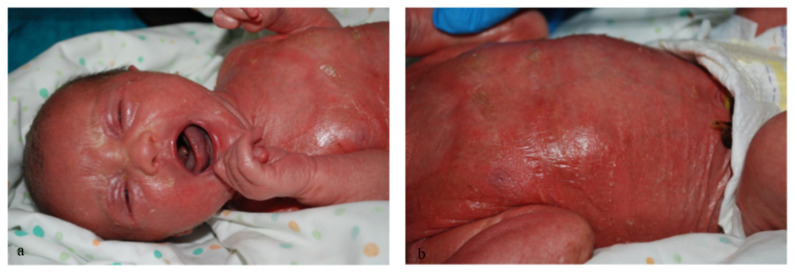
Patient 2 at birth: (**a**) craniofacial features of Collodion Baby Syndrome (CBS), including ectropion and eclabium. (**b**) The skin is covered by a tight, shiny collodion membrane involving nearly the entire body surface, with a taut, parchment-like appearance and early desquamation and fissures over the trunk and extremities.

A female newborn was delivered at 39 + 0 weeks of gestation by cesarean section due to non-reassuring cardiotocography during labor in an otherwise uncomplicated pregnancy. She was the first child of healthy, non-consanguineous parents, with no relevant family history of genetic disorders. Maternal screening for TORCH infections and vaginal and rectal swabs for Group B Streptococcus were negative, and obstetric ultrasound examinations during pregnancy demonstrated appropriate fetal growth and development. At birth, the newborn showed good postnatal adaptation (Apgar scores of 9 and 10 at 1 and 5 min, respectively) and normal anthropometric parameters: birth weight 2920 g (28th percentile), length 49 cm (50th percentile), and head circumference 35 cm (86th percentile), according to Italian INeS growth charts [7]. Physical examination revealed marked skin dehydration with a shiny, translucent membrane covering nearly the entire body surface, most evident on the face, trunk, and limbs, associated with thick whitish desquamating plaques over the body, along with mild ectropion and eclabium (Figure 4a,b).

No other abnormalities were observed. Due to the suspicion of Collodion Baby Syndrome (CBS), the newborn was immediately transferred to the NICU of the Maggiore Hospital in Bologna, Italy. On admission, a complete neurological examination showed normal tone and reflexes, with no additional abnormalities detected. To rule out associated congenital malformations, imaging studies including cerebral, cardiac, and abdominal ultrasound were performed and yielded normal results, as did audiological and ophthalmological assessments. Given the clinical suspicion of congenital ichthyosis, targeted next-generation sequencing (NGS) was performed using a 56-gene panel associated with genodermatoses (Table 2).

The analysis identified two heterozygous variants in the *CYP4F22* gene (NM_173483.4): c.1084C>T p.(Arg362Ter), classified as pathogenic, and c.543_545del p.(Ile181del), classified as a variant of uncertain significance (VUS). Parental segregation analysis was refused due to personal reasons. Consequently, the allelic phase of the two variants could not be established, and the presence of a biallelic disease-causing genotype remains unconfirmed. Upon NICU admission, the infant was placed in a humidified incubator with controlled temperature (36–37 °C) and humidity (70%), to prevent transepidermal water loss and hypernatremic dehydration. Enteral feeding with formula milk was initiated during the first 24–48 h, with subsequent integration of breastfeeding using a sterile medical-grade polyethylene wrap as a protective interface between the mother and the infant. Following dermatological consultation, topical treatment with emollient creams was applied every 4–6 h, along with lubricating eye drops for ectropion management. Given the stable clinical condition, progressive weight gain, and improvement in skin integrity, incubator humidity was gradually reduced starting from the second day of life, with progressively increased exposure to the external environment. Complete transition out of the humidified setting was achieved by day 15, without recurrence of desquamation, dryness, or skin breakdown. The infant was discharged on day 20 in good clinical condition, with dermatological and genetic follow-up arranged.

**Figure 5:**
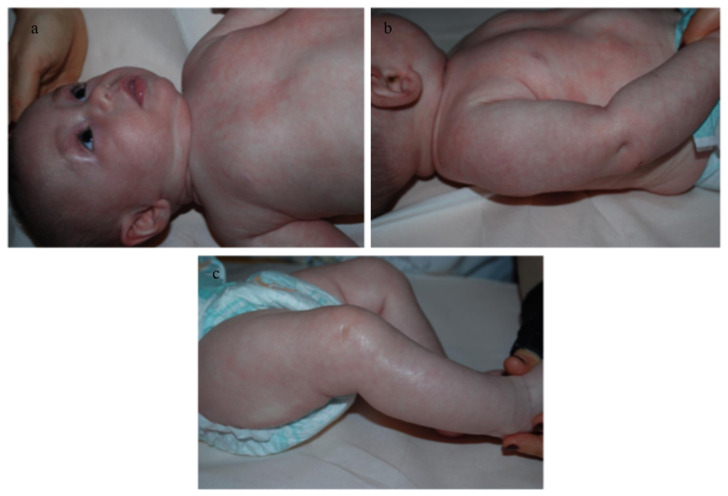
Patient 2 at age 4 months. (**a**) Facial examination shows complete resolution of the collodion membrane with normal skin appearance, except for minimal erythema and dryness on the upper eyelids. (**b**) Trunk and (**c**) lower extremities demonstrating complete healing with well-hydrated, normal skin and no residual abnormalities.

At the 4-month dermatological evaluation, the infant was in good clinical condition and showed appropriate growth parameters, with a weight of approximately 6300 g (50th percentile), length of 62 cm (50th percentile), and head circumference of 41 cm (70th percentile), indicating normal growth velocity and adequate nutritional status [8]. The dermatological findings had almost completely resolved, with only minimal areas of erythema and dryness on the upper eyelids. No other cutaneous abnormalities were observed (Figure 5a–c).

#### 2.2.1. Genetic Analysis

Genomic DNA extracted from peripheral blood of the proband was analyzed using NGS with targeted exon sequencing of 56 genes associated with congenital ichthyosis and genodermatoses. The analysis was performed using the Genodermatosi 2020 panel (Thermo Fisher Scientific, Waltham, MA, USA), covering a total target size of 322.6 kb and 1703 amplicons. Sequencing was carried out on an Ion Torrent S5 platform (Thermo Fisher Scientific, Waltham, MA, USA). All detected variants were interpreted according to the American College of Medical Genetics and Genomics (ACMG) guidelines [11].

This analysis identified two heterozygous variants in the *CYP4F22* gene (NM_173483.4). The first variant, c.1084C>T p.(Arg362Ter), is a nonsense mutation predicted to introduce a premature termination codon at position 362, likely resulting in a truncated protein or nonsense-mediated mRNA decay. According to ClinVar and VarSome databases [13], this variant is classified as pathogenic (ACMG class 5) and has a reported allele frequency of approximately 1/121,000 in population databases (gnomAD v4.0) [17]. The second variant, c.543_545del p.(Ile181del), is an in-frame deletion of three nucleotides resulting in the loss of an isoleucine residue at position 181. This variant is extremely rare, with an allele frequency of approximately 1/1,460,000 in gnomAD v4.0 [17], and is currently absent from ClinVar. According to ACMG criteria [11], it is therefore classified as a variant of uncertain significance (VUS; class 3). Parental segregation analysis could not be performed because genetic testing was declined for personal reasons. Therefore, although the presence of two heterozygous variants in *CYP4F22* suggests a possible compound heterozygous genotype, the allelic phase (cis/trans configuration) could not be formally established, representing a limitation of this study.

A bioinformatic analysis was performed to assess the potential functional impact of the c.543_545del p.(Ile181del) variant. The deleted residue is located within a highly conserved region of the CYP4F22 protein across multiple species. *In silico* prediction tools, including the online versions of PROVEAN and MutationTaster available in December 2025, suggested that this alteration may exert a deleterious effect on protein structure and enzymatic activity (see the dedicated section below). *CYP4F22* encodes a membrane-bound cytochrome P450 enzyme involved in epidermal lipid metabolism and skin barrier formation; therefore, alterations affecting conserved residues may impair its catalytic function. Although the structural domain affected by p.(Ile181del) has not been functionally characterized, its high evolutionary conservation suggests a potential role in protein stability or membrane-associated folding.

According to ACMG criteria (PM2 and PP3) [11], the variant does not yet fulfill sufficient criteria to be classified as likely pathogenic and therefore remains a variant of uncertain significance (ACMG class 3). However, its occurrence in a patient carrying another pathogenic variant in *CYP4F22*, and presenting with a phenotype consistent with autosomal recessive congenital ichthyosis, is compatible with a potential contribution to the observed phenotype, although no conclusion regarding pathogenicity can be drawn without segregation studies and functional validation.

#### 2.2.2. Bioinformatic Analysis

For the bioinformatic analysis, computational predictions were retrieved using the VarSome search engine and the Ensembl database, integrating several *in silico* tools commonly used for variant interpretation, including SIFT, PolyPhen-2, Combined Annotation Dependent Depletion (CADD), Rare Exome Variant Ensemble Learner (REVEL), and MetaLR [24,25,26,27,28]. These tools were used to evaluate the potential functional impact of the identified variants based on evolutionary conservation, predicted effects on protein structure, and possible impairment of enzymatic activity. Additional details of the bioinformatic predictions are provided in the Supplementary File S2.

## 3. Discussion and Literature Review

**Figure 6:**
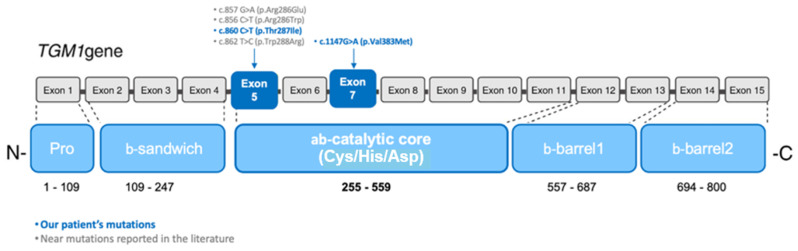
(Modified by Ebrahimi Samani S. et al., 2024 [30]). Schematic representation of the human keratinocyte *TGM1* gene and protein domains. Functional domains, including the catalytic core and other critical regions, are indicated, highlighting clustering of pathogenic variants. The maternally inherited variant, previously reported as pathogenic, is located in exon 7 (c.1147G>A; p.Val383Met). The paternally inherited variant, c.860C>T p.(Thr287Ile), not previously described as pathogenic, is located in exon 5, in close proximity to other variants (p.Trp288Arg, p.Arg286Gln, p.Arg286Trp) known to have deleterious effects.

Autosomal recessive congenital ichthyoses (ARCIs) are lifelong disorders of cornification characterized by generalized scaling and variable erythema, typically presenting at birth or in early infancy. ARCIs belong to the group of non-syndromic ichthyoses, which also include some of the most severe and potentially life-threatening phenotypes, such as harlequin ichthyosis (HI), lamellar ichthyosis (LI), and nonbullous congenital ichthyosiform erythroderma (CIE). Minor variants include self-improving collodion ichthyosis (SICI) and bathing suit ichthyosis (BSI) [2]. These phenotypes exist along a clinical *continuum*, and careful characterization of affected patients is essential to refine prognosis and guide management [3]. While some forms of congenital ichthyosis can be recognized immediately at birth, others require longitudinal observation for accurate and definite diagnosis. LI, as observed in our first patient, often presents with a collodion membrane at birth, which evolves into large, plate-like, whitish to brown scales with generalized distribution. Common associated findings include ectropion, eclabium, and scarring alopecia affecting the scalp and eyebrows. Mutations in the *TGM1* gene are strongly associated with ARCI and were identified, indeed, in our Patient 1. In approximately 80–90% of cases with *TGM1* variants, neonates are born as collodion babies, frequently presenting with severe ectropion. Around 90% of cases evolve into LI, whereas the remainder manifest as CIE [6]. The *TGM1* gene, located on chromosome 14q12, encodes transglutaminase-1, essential for the formation of the cornified cell envelope in the *stratum corneum*. This enzyme catalyzes protein crosslinking and attachment of polyamines to structural proteins; disruption of these processes underlies the impaired skin barrier and clinical manifestations in CI [29,30,31]. Both missense and truncating biallelic mutations in *TGM1* are associated with LI (MIM #242300). In our proband, molecular analysis identified one previously reported pathogenic variant and one novel likely pathogenic variant in *TGM1*. Bioinformatic analyses suggested deleterious effects for both variants, consistent with *TGM1* intolerance to missense and truncating variants (gnomAD v4.1) [17]. The maternal variant, p.(Val383Met), is reported in unrelated patients with CI, either in homozygosity or compound heterozygosity, and is classified as pathogenic or likely pathogenic according to ACMG criteria [11,32,33]. The paternal variant, p.(Thr287Ile), has not been previously reported, but affects a residue located in a mutational hotspot. *In silico* predictions provide moderate support for a deleterious effect, and pathogenic substitutions affecting adjacent residues (p.Trp288Arg, p.Arg286Gln, p.Arg286Trp) have been reported, supporting the biological relevance of this region, although the pathogenic effect of p.(Thr287Ile) remains inferential in the absence of functional studies. Figure 6 schematically depicts the location of the two variants in the *TGM1* gene in relation to previously reported pathogenic changes. The clustering of variants within functionally relevant regions further supports their pathogenic role.

Genotype–phenotype correlations in *TGM1*-related disease are complex and cannot be explained solely by zygosity status. Current evidence suggests that disease severity is more strongly influenced by the functional consequences of the variants, particularly the presence of premature termination codons, loss-of-function alleles, and mutations affecting critical catalytic or structural domains of transglutaminase-1. Variants predicted to retain residual enzymatic activity may be associated with milder phenotypes or partial clinical improvement [34,35]. In our Patient 1, both variants are missense changes, and no truncating allele was identified, a finding that may have contributed to the relatively favorable clinical course observed during follow-up.

A structured literature review was performed using PubMed and Scopus databases. Searches were conducted between January and February 2026 and included publications from January 2000 to December 2025 for *TGM1* and from January 2011 to December 2025 for *CYP4F22*. Search terms included combinations of “congenital ichthyosis”, “autosomal recessive congenital ichthyosis”, “lamellar ichthyosis”, “collodion baby”, “*TGM1*”, “*CYP4F22*”, “newborn”, “genotype”, and “phenotype”. Eligible studies were original case reports or case series reporting pediatric patients with molecularly characterized *TGM1*- or *CYP4F22*-related disease. Reviews, conference abstracts, letters without clinical data, and duplicate reports were excluded. When overlapping cohorts were identified, the most comprehensive publication was retained. Phenotypic information was extracted from the original reports and summarized descriptively. Owing to the rarity of the conditions and the heterogeneity of reporting, a formal systematic review or PRISMA-based meta-analysis was not performed. Table 3 summarizes the main phenotypic and genotypic features of *TGM1*-nEDD subjects reported in the literature compared to our Patient 1.

A broad spectrum of *TGM1* mutations—including nonsense, missense, splicing, and frameshift—have been described. Most cases are diagnosed at birth, often as collodion babies, though delayed diagnoses are reported. Clinical outcomes are variable, with many patients showing improvement during the first year of life, but long-term treatment is frequently needed to control scaling and prevent complications [45,47,53]. Our Patient 1, as well, achieved satisfactory disease control using topical emollients alone.

The current second case describes a female newborn presenting with clinical features highly suggestive of *CYP4F22*-related nEDD, and carrying two heterozygous *CYP4F22* variants. This gene encodes a membrane-bound cytochrome P450 enzyme involved in the ω-hydroxylation step required for acylceramide synthesis. Acylceramides are critical lipid components of the *stratum corneum* lipid lamellae, which are essential for epidermal barrier integrity, and also influence lamellar body secretion and permeability barrier formation [54,55]. Recent reviews have highlighted how defects in cornified envelope formation, epidermal lipid processing, and lamellar body secretion converge to impair epidermal barrier integrity in ARCI. Disruption of these interconnected pathways results in increased transepidermal water loss, altered keratinocyte differentiation, and chronic inflammation, providing a mechanistic framework linking diverse genetic defects to overlapping clinical phenotypes [56,57]. Pathogenic variants result in impaired epidermal barrier function and the clinical phenotype of *CYP4F22*-nEDD (formerly ARCI-5, OMIM 604777); *CYP4F22*-related disease typically consists of a persistent ichthyosis phenotype, although some degree of phenotypic variability has been reported, and progression toward a milder presentation, including self-improving collodion ichthyosis, cannot be entirely excluded, although this appears to be uncommon. Our Patient 2 showed complete clinical resolution within the first months of life under topical emollient therapy. The clinical course is consistent with a self-improving collodion ichthyosis (SICI)-like evolution within the ARCI spectrum, as previously reported in *CYP4F22*-related cases. Genetic analysis identified, indeed, a truncating variant of the gene, c.1084C>T p.(Arg362Ter), with a second rare *CYP4F22* VUS, c.543_545del p.(Ile181del). While the truncating allele is clearly pathogenic, the in-frame deletion had not been previously reported. Evolutionary conservation analyses showed that Ile181 is located within a highly conserved region of CYP4F22. Computational predictions were considered only as supportive evidence according to ACMG recommendations. Although the reading frame is maintained, deletion of this conserved residue may compromise protein structure or enzymatic activity. The presence of the p.(Ile181del) variant together with a pathogenic *CYP4F22* allele and a compatible phenotype raises the possibility of a contributory role; however, this remains a hypothesis because the variant is currently classified as a VUS and segregation analysis was unavailable. Indeed, the pathogenicity of the in-frame deletion cannot be definitively established in the absence of functional studies. Recent research indicates that *CYP4F22*-related ARCI-5 generally presents with milder severity compared to *TGM1* or *ABCA12* mutations, consistent with our patient’s spontaneous improvement within the first months of life [58,59,60,61,62,63]. Several studies suggest that the clinical phenotype of *CYP4F22*-related disease is influenced primarily by the functional impact and location of the variants rather than by homozygosity or compound heterozygosity alone. Truncating variants introducing premature stop codons, particularly when affecting essential catalytic regions or occurring in both alleles, are generally associated with more persistent and severe ichthyosis. Conversely, the presence of at least one non-truncating allele may permit residual protein function and contribute to a milder phenotype or spontaneous improvement. With regard to the literature review (2011–2025) focusing on pediatric patients with homozygous or compound heterozygous *CYP4F22* variants, Table 4 summarizes the main phenotypic and genotypic features reported in the literature compared to our Patient 2.

Assessment of disease severity in the literature review was based on the clinical information provided by the original reports. Cases were considered relatively severe when persistent generalized ichthyosis, extensive hyperkeratosis, marked ectropion/eclabium, palmoplantar keratoderma, alopecia, or long-term need for systemic therapy were reported. Conversely, cases showing substantial spontaneous improvement, minimal residual scaling, or complete resolution during infancy were considered milder. Because of the heterogeneity of published reports and the lack of standardized severity scores in most studies, these comparisons should be interpreted cautiously and regarded as descriptive rather than quantitative. Based on these premises, our research found that the p.(Arg362Ter) variant has been previously reported in association with ARCI and is consistently classified as pathogenic, in line with the expected loss-of-function mechanism described for *CYP4F22*. Similar truncating variants, particularly in homozygous state, are generally associated with more severe and persistent phenotypes, as reflected in several cases summarized in Table 4. Regarding the second p.(Ile181del) variant, comparison with cases reported in the literature suggests that missense or in-frame variants affecting conserved residues may allow for partial residual enzyme activity, contributing to phenotypic attenuation. Notably, several patients with compound heterozygous *CYP4F22* variants—including at least one non-truncating allele—showed significant clinical improvement over time or even near-complete resolution, as observed also in our case. This contrasts with reports of patients harboring biallelic truncating variants, who more frequently develop persistent ichthyosis. Overall, the genotype observed in our Patient 2 appears consistent with previously described genotype–phenotype correlations in ARCI-5, supporting the hypothesis that the presence of at least one partially functional allele may mitigate disease severity. The identification of the novel p.(Ile181del) variant adds a previously unreported *CYP4F22* sequence variant that may be relevant for future genotype interpretation, although its pathogenic significance remains uncertain. Further segregation and functional studies will be required to clarify its clinical significance.

Although the clinical presentations observed in our patients fall within the known phenotypic spectrum of *TGM1*- and *CYP4F22*-related disease, these cases are noteworthy because they harbor previously unreported variants and provide additional evidence regarding the interpretation of rare sequence changes in congenital ichthyosis, as occur when implementing NGS for other rare diseases [67,68,69,70,71,72]. Overall, comparison with previously reported cases suggests a possible genotype–phenotype correlation, whereby truncating variants tend to be associated with more persistent disease, whereas the presence of at least one non-truncating allele may contribute to a milder or self-resolving phenotype, potentially through partial restoration of epidermal barrier function. The integration of literature comparisons (Table 3 and Table 4) and schematic visualization of gene mutations (Figure 4) enhances the interpretation of variant pathogenicity, and supports more accurate prognostic stratification. Functional studies were not performed, representing a limitation of the present report; however, the integration of clinical findings, segregation analysis (when available), evolutionary conservation, and in silico predictions provides supportive evidence for variant interpretation.

## 4. Conclusions

Our report highlights the challenges in managing patients with nEDDs and underscores the importance of comprehensive, multidisciplinary care from birth. Some forms of ichthyosis, such as classic *TGM1*-nEDD presenting with a lamellar ichthyosis phenotype, can be recognized postnatally by their characteristic collodion baby presentation and persistent lamellar scaling. Other subtypes, including *CYP4F22*-nEDD (ARCI-5) due to *CYP4F22* variants, may present with a collodion membrane at birth but show progressive spontaneous improvement within the first months of life, reflecting a milder phenotype along the ARCI spectrum.

Targeted next-generation sequencing (NGS) has proven invaluable for diagnosing these genetically heterogeneous conditions, particularly when phenotypic overlap complicates clinical assessment [53,71]. In our patients, NGS enabled identification of both previously described pathogenic variants, i.e., *TGM1* c.1147G>A p.(Val383Met) and *CYP4F22* c.1084C>T p.(Arg362Ter), and the novel variants *TGM1* c.860C>T p.(Thr287Ile) and *CYP4F22* c.543_545del p.(Ile181del), supporting precise molecular characterization. For Patient 2, however, the absence of segregation analysis prevents definitive molecular confirmation of autosomal recessive inheritance, and should be considered when interpreting the molecular findings. When available, *in trio* molecular testing may allow for accurate genetic counselling, including identification of heterozygous carriers within the family and enabling reproductive planning in future pregnancies. Finally, early molecular confirmation of diagnosis informs prognosis and guides long-term management strategies, including dermatologic care and monitoring for complications, thereby improving patient outcomes and family quality of life, as reported for other congenital diseases [68,70,73]. The principal novelty of this report lies in the identification of previously unreported *TGM1* and *CYP4F22* sequence variants. Both patients exhibited clinical manifestations broadly consistent with previously described *TGM1*- and *CYP4F22*-associated disease. Recognizing the spectrum of ARCI phenotypes—from persistent lamellar ichthyosis to self-improving ARCI-5—is essential for appropriate clinical management and family counselling.

## Figures and Tables

**Table 1:** Genes associated with ichthyosis and erythrokeratoderma included in the next-generation sequencing panel used for Patient 1.

Gene	RefSeq
*AAGAB*	NM_024666.5
*ABCA12*	NM_173076.3
*ABHD5*	NM_016006.6
*ALDH3A2*	NM_000382.3
*ALOX12B*	NM_001139.3
*ALOXE3*	NM_012628.3
*AQP5*	NM_001651.4
*ASPRV1*	NM_152792.4
*CARD14*	NM_001366385.1
*CAST*	NM_001750.7
*CDSN*	NM_001264.5
*CERS3*	NM_001378789.1
*CLDN1*	NM_021101.5
*CSTA*	NM_005213.4
*CYP4F22*	NM_173483.4
*DSC2*	NM_024422.6
*DSG1*	NM_001942.4
*DSP*	NM_004415.4
*ENPP1*	NM_006208.3
*FLG*	NM_002016.2
*GJA1*	NM_000165.5
*GJB2*	NM_004004.6
*GJB3*	NM_024009.3
*GJB4*	NM_153212.3
*GJB6*	NM_001110219.3
*JUP*	NM_002230.4
*KDSR*	NM_002035.4
*KRT1*	NM_006121.4
*KRT10*	NM_000421.5
*KRT14*	NM_000526.5
*KRT16*	NM_005557.4
*KRT17*	NM_000422.3
*KRT6A*	NM_005554.4
*KRT6B*	NM_005555.4
*KRT6C*	NM_173086.5
*KRT9*	NM_000226.4
*NIPAL4*	NM_001099287.2
*PIGL*	NM_004278.4
*PNPLA1*	NM_001374623.1
*POMP*	NM_015932.6
*RHBDF2*	NM_001005498.4
*RSPO1*	NM_001242908.2
*SERPINB7*	NM_003784.4
*SLC27A4*	NM_005094.4
*SLURP1*	NM_020427.3
*SNAP29*	NM_004782.4
*SPINK5*	NM_006846.4
*SREBF1*	NM_004176.5
*ST14*	NM_021978.4
*STS*	NM_001320752.2
*SULT2B1*	NM_177973.2
*TAT*	NM_000353.3
***TGM1***	**NM_000359.3**
*TGM5*	NM_201631.4

**Table 2:** Genes included in the targeted next-generation sequencing panel used for Patient 2.

Gene	RefSeq
*ABCA12*	NM_173076.3
*ABHD5*	NM_016006.6
*ALDH3A2*	NM_000382.4
*ALOX12B*	NM_001139.3
*ALOXE3*	NM_012628.3
*CDSN*	NM_001264.5
*CERS3*	NM_001378789.1
*CHST8*	NM_001157.4
*CLDN1*	NM_021101.5
*COL17A1*	NM_000494.4
*COL7A1*	NM_000088.3
*CSTA*	NM_001262.4
***CYP4F22***	**NM_173483.4**
*DSG1*	NM_001942.4
*DSP*	NM_004415.4
*DST*	NM_015548.4
*EBP*	NM_006579.4
*EXPH5*	NM_020102.6
*FERMT1*	NM_015568.4
*GJB2*	NM_004004.5
*GJB3*	NM_024009.3
*GJB4*	NM_153212.3
*ITGA3*	NM_002204.4
*ITGA6*	NM_000210.3
*ITGB4*	NM_000213.3
*JUP*	NM_002230.4
*KRT1*	NM_006121.4
*KRT10*	NM_000421.5
*KRT14*	NM_000526.5
*KRT16*	NM_005557.4
*KRT17*	NM_000422.3
*KRT2*	NM_000423.4
*KRT5*	NM_000424.4
*KRT6A*	NM_005554.4
*KRT6B*	NM_005555.4
*KRT6C*	NM_173086.5
*KRT9*	NM_000226.4
*LAMA3*	NM_000227.4
*LAMB3*	NM_000228.3
*LAMC2*	NM_005562.4
*LIPN*	NM_017462.3
*LOR*	NM_000427.3
*MBTPS2*	NM_022041.3
*NIPAL4*	NM_001099267.2
*NSDHL*	NM_015922.6
*PEX7*	NM_000288.4
*PHYH*	NM_006384.4
*PKP1*	NM_006214.4
*PLEC*	NM_000445.5
*PNPLA1*	NM_001374263.1
*POMP*	NM_015932.6
*PORCN*	NM_203475.3
*RECQL4*	NM_004260.4
*SLC27A4*	NM_005094.4
*SPINK5*	NM_006846.4
*ST14*	NM_021978.4
*STS*	NM_000351.2
*SUMF1*	NM_182760.3
*TGM1*	NM_000359.3
*TGM5*	NM_201631.4

**Table 3:** Comparison between our Patient 1 and those previously described in the literature affected by congenital ichthyosis due to *TGM1* variants.

Authors, Year, Country	Mutation in *TGM1* Gene	Sex	Gestational Age	Onset/Age at Diagnosis	Clinical Features	Treatment	Outcome
Shevchenko YO et al. [36], 2000, USA	sp/p.(Arg389Pro) (compound heterozygous)	F	n.r.	n.r./5 years	Ectropion, palmoplantar hyperkeratosis, platelike scale	PG, tT	n.r.
Shevchenko YO et al. [36], 2000, USA	sp/p.(Arg389Pro) (compound heterozygous)	M	n.r.	n.r./4 years	Ectropion, palmoplantar hyperkeratosis, plate-like scale, alopecia	PG, tT	n.r.
Shevchenko YO et al. [36], 2000, USA	sp/p.(Arg389Pro) (compound heterozygous)	M	n.r.	n.r./2 years	Ectropion, palmoplantar hyperkeratosis	PG, tT	n.r.
Shevchenko YO et al. [36], 2000, USA	sp/p.(Arg760Ter) (compound heterozygous)	M	n.r	n.r./9 years	Ectropion, eclabium, palmoplantar hyperkeratosis, platelike scale, alopecia	PG, LA, tT	n.r.
Cserhalmi-Friedman PB et al. [37], 2001, USA	p.(Gly278Arg) p.(Arg286Gln) (compound heterozygous)	n.r.	n.r.	n.r./n.r.	n.r.	n.r.	Mild erythema, large scale
Shawky RM et al. [38], 2004, Egypt	p.(Arg142His) (homozygous)	M	n.r.	At birth/2.5 years	At birth: scaling of skin, dark scales affecting the entire body	VA, U	Palmo-plantar hyperkeratosis
Shawky RM et al. [38], 2004, Egypt	p.(Arg142His) (homozygous)	M	Term	At birth/2 years	Collodion baby at birth	SPB, VA, U, AHA, PG	Ectropion, palmo-plantar hyperkeratosis, scales
Shawky RM et al. [38], 2004, Egypt	p.(Arg142His) (homozygous)	F	n.r.	At birth/12 years	Collodion baby at birth	VA, U, AHA	Dry rough scaly skin covering the whole body with dark areas over the elbows and knees
Mazereeuw-Hautier J et al. [39], 2009, United Kingdom	p.(Val359Met) p.(Arg396His)	F	Term	At birth	Acral self-healing Collodion Baby. The two last phalanges of fingers and toes were embedded in a collodion membrane	n.r.	Skin abnormalities healed within three weeks. Currently, 7 years old, completely normal skin picture
Mazereeuw-Hautier J et al. [39], 2009, United Kingdom	c.1922_1926 + 2delGGCC p.(Arg396His) TGT (deletion mutation)	F	Term	At birth	Classic LI: generalized large dark scales all over the body	n.r.	n.r.
Esposito G. et al. [40], 2009, Italy	p.(Thr263X) (homozygous nonsense mutation)	F	n.r.	At birth	Collodion baby and respiratory distress	n.r.	At age 3 years: severe non-erythrodermic LI, large, lamellar scales over the entire body. Ectropion and nail dystrophy. Hyperkeratotic palms and soles. Scarring alopecia
Cao et al. [41], 2009, China	c.607C>T (p.Q203X) in exon 4 (homozygous nonsense mutation)	M	Term	At birth	Collodion membrane	No therapy	Ectropion, alopecia, hypohidrosis and palmoplantar hyperkeratosis with large brown plate-like scales on trunk and limbs
Cao et al. [41], 2009, China	p.(Arg687His) (Homozygous mutation)	M	n.r.	Within few days from birth	His skin started peeling 5 days after birth	n.r.	Large brown plate-like scales on the trunk, limbs and forehead with palmoplantar hyperkeratosis and hypohidrosis
Cao et al. [41], 2009, China	c.760G>A in exon 5, p.(D254N) G → T transversion at the first nucleotide of intron 4 (IVS4 + 1G → T) (Compound heterozygous for one missense mutation and one splicing mutation)	M	Term	At birth	Collodion Baby	n.r.	Large brown scales over his whole body with ectropion, alopecia, hypohidrosis and palmoplantar hyperkeratosis
Rodríguez-Pazos L et al. [42], 2009, Spain	c.2278C>T p.(R760X) (Homozygous nonsense mutation)	M	Term	At birth	Collodion Baby without other congenital anomalies	n.r.	4-month-old: body covered with large, white, plate-like scales. Ectropion and eclabium. Peripheral alopecia on the scalp
Hackett BC et al. [34], 2010, United Kingdom	c.944G>T p.(Arg315Leu) in exon 6 (Missense mutation) c.1331dupA p.(Arg445fs) in exon 9 (Frameshift mutation)	M	Late preterm	At birth	Collodion membrane with eclabion and ectropion at birth.	n.r.	During the first month of life, lamellar scaling developed on the trunk with complete sparing of limbs and face
Hackett BC et al. [34], 2010, United Kingdom	c.1264A>T p.(Lys422X) in exon 8 (Nonsense mutation) c.1352G>T p.(Gly451Val) in exon 9 (Missense mutation)	n.r.	Late preterm	At birth	Collodion membrane at birth	Regular syringing of the ears due to excessive desquamation within the external auditory canal.	No evidence of lamellar scaling apart from mild scaling in the axillae
Hackett BC et al. [34], 2010, United Kingdom	c.919C>G p.(Arg307Gly) in exon 6 (Homozygous missense mutation)	Twins/gender not reported	Late preterm	At birth	A collodion membrane was present at birth in both patients	No therapy	Follow-up at 14 years revealed a self-healing phenotype with no sequelae
Fachal L et al. [43], 2012, Spain	c.984+1G>A (Homozygous splicing mutation)	F	Preterm	At birth	Collodion membrane without other congenital anomalies	Intermittent courses of acitretin at doses of 0.5–1 mg/kg per day since the age of three years	Generalized whitish plate-like scales on the whole body, severe ectropion, hyperkeratosis of palms and soles. Peripheral alopecia on the scalp
Al-Naamani A et al. [44], 2013, Oman	c.832G>A in exon 5 p.(Gly278Arg) (Homozygous missense mutation)	M	n.r.	At birth	Collodion membrane, ectropion	Acitretin (intermittently)	Mild generalized scaling during childhood
Al-Naamani A et al. [44], 2013, Oman	c.832G>A in exon 5 p.(Gly278Arg) (Homozygous missense mutation)	n.r.	n.r.	At birth	Collodion baby, covered with a thick taut skin membrane, ectropion, eclabium, and flattening of nose and ears	Initial treatment: acitretin for 2 weeks, amoxicillin-clavulanate antibiotic and liquid paraffin. Current treatment: emollients and eye lubricants along with low-dose acitretin (10 mg on alternate days)	Currently, classic features of LI with brownish fish-like scales on the skin accentuating the flexures, associated with ectropion and alopecia at the temporal scalp
Al-Naamani A et al. [44], 2013, Oman	c.1187G>A in exon 8 p.(Arg396His) (Homozygous missense mutation)	n.r.	n.r.	At birth	Collodion membrane, ectropion	Acitretin and emollient (frequent use)	Mild generalized scaling during childhood
Sugiura K et al. [35], 2013, Japan	c.232C>T p.(Arg78X) c.2077C>T p.(Leu693Phe) (compound heterozygous)	M	Term	At birth	Collodion membrane with eclabium	No therapy	A skin biopsy from the lesion on the trunk at 18 days after birth showed mild hyperkeratosis with normal granular layers
Park SH et al. [45], 2013, South Korea	c.424C>T on exon 3, p.(R142C). Missense mutation c.1160-2A>C (IVS7-2A>C). Splicing mutation that results in skipping exon 8 (Compound heterozygous)	M	Term	At Birth	Collodion baby	At birth: artificial lubricant and antibiotic ointment were applied continuously to prevent exposure keratopathy and secondary infection. Then: emollients and tT	6-day-old: platelike hyperkeratotic scales covering the whole body surface. Upper and lower ectropion with hyperkeratosis and lagophthalmos
Benmously-Mlika R et al. [46], 2014, Tunisia	p.(Ile304Phe) (Homozygous)	F	n.r.	At birth	Collodion baby, bilateral ectropion and eclabion	Emollients and keratolytics	At age 3 months, brownish scaling restricted to the bathing suit area. At age 3 years, the scales remained limited to the bathing suit area
Tanahashi et al. [47], 2014, Japan	c.919C>T p.(Arg307Trp) c.2180G>A p.(Arg727Gln) (compound heterozygous)	F	Term	At birth	Collodion baby	No therapy	Her skin spontaneously healed by the age of 2 months
Suga et al. [48], 2015, Japan	c.211C>T p.(R71*) in the N-terminal domain c.508+668C>T p.(N171Tfs*45) in the b-sandwich domain	M	Term	At birth	Collodion baby	Topical lotions and creams containing hyaluronic acid, petrolatum and/or ointment containing 0.5% vitamin A oil. Ophthalmic solution.	Ectropion, desquamations of the scalp, lower legs covered by large, thick, brownish scales, his trunk and arms were dry with polygonal, small, brownish scales. Palms and soles showed hyperkeratosis. He grew within −1 standard deviation of height and weight
Bastaki et al. [49], 2017, United Arab Emirates	c.1085T>G in exon 7 p.(Leu362Arg) (Homozygous missense mutation)	F	Term	At birth	Collodion baby	Symptomatic local skin applications, local eye drops	At 1 year and 8 months generalized large thick skin plates with cracks and thin membranes in-between. Peeling of skin, ectropion, and eclabium
Zaouak A et al. [50], 2019, Tunisia	c.788G>A p.(Try263X) (Homozygous nonsense mutation)	M	n.r.	At birth	Collodion baby At 13 years old: lamellar scales covering his entire body, palmoplantar keratoderma with hyperlinearity, ectropion. History of progressive knee deformity and difficulty in walking (due to vitamin D rickets)	Vitamin D and calcium supplementation, along with regular sun exposure and a diet rich in vitamin D and calcium	After 3 months, improvement of calcium and vitamin D levels and of bone deformities
Diep QM et al. [51], 2020, Vietnam	c.968G>A p.(Arg323Gln) c.1756C>T p.(Gln586Stop)	F	Term	At birth	At birth: Collodion baby. During the next few weeks, the collodion membrane was replaced by a layer of hyperkeratosis with platelike adherent scales on the trunk and limbs	Petrolatum, a bland emollient	At 4 months, nearly normal skin, apart from occasion patches of dry, rough cutaneous areas on her back
Guo et al. [52], 2025, USA	c.425G>A p.(Arg142His) c.420A>G p.(Ile140Met) (compound heterozygous)	F	Term	At birth	The skin was erythematous and shiny, covered by a tense, transparent, plastic-like membrane. Notable desquamation on the limbs and buttocks, with erosions in the groin area. Dry scales in the back. Bilateral ectropion, stiff ears, eclabium	Mixture of petroleum jelly and medical moisturizing cream. Erythromycin ointment applied to the areas of erosion	After 15 days of treatment, the infant’s condition stabilized, and by discharge (after 30 days), the skin returned to normal, except for the head and extremities
Patient 1 (2025)	p.(Val383Met) p.(Thr287Ile) (compound heterozygous)	F	Term	At birth	parchment-like skin, erythematous body, some cracked and macerated areas. Eclabium, ectropion, joints stiffness	tT: white vaseline, FA, vitamin enriched lipid-replenishing and hydrating oil	At age 7 months, the patient showed adequate growth. Dysepithelized skin areas on the back, right flank, inguinal region and under the axilla

AHA: alpha hydroxy acid; F: female; LA: lactic acid; M: male; n.r.: not reported; PG: propylene glycol; sp: splice-site mutations; SPB: sodium phenobarbitone; tT: topical treatment; U: urea; VA: oral vitamin A.

**Table 4:** Comparison between our Patient 2 and those previously described in the literature affected by congenital ichthyosis due to *CYP4F22* variants.

Authors, Year, Country	Mutation in *CYP4F22* Gene	Sex	Gestational Age	Onset/Age at Diagnosis	Clinical Features	Treatment	Outcome
Feng C et al. [63], 2016, China	c.844C>T p.(R282W) (Homozygous mutation)	M	n.r.	At birth	Mild erythroderma	n.r.	Whitish-grey scaling predominantly affecting the limbs and lower trunk. Hyperlinearity and hyperkeratosis of the palms and soles
Feng C et al. [63], 2016, China	c.466>T p.(R156C) (Homozygous mutation)	M	n.r.	At birth	Collodion baby without ectropion or eclabium	n.r.	Whitish-grey scaling predominantly affecting the limbs and lower trunk. Hyperlinearity and hyperkeratosis of the palms and soles
Gruber R et al. [61], 2017, England	c.549+5G>C (Homozygous splice-site mutation)	F	Late preterm	At birth	Collodion baby with contractures of the great joints and palmoplantar hyperlinearity. No eclabion and ectropion	Topical treatment was carried out with emollient ointments	At age of 6 months fine scaling of the skin with erythroderma. Complete resolution by the age of 9 months
Dumenigo et al. [58], 2022, Cincinnati—USA	Autosomal recessive, heterozygous CYP4F22-related congenital ichthyosis	M	n.r.	At birth	Thick, yellow-brown, shiny membrane, underlying erythema	Topical treatment with emollients	At 3 months of age overall improvement of erythema and scaling.
Noguera-Morel L et al. [60], 2016, Spain	c.728G>A p.(Arg243His) (homozygous missense mutation)	F	Late preterm	At birth	Collodion baby	n.r.	At 6 months of age s almost no signs of disease except for minimal facial erythema, very mild scaling, especially on the axillae, and hyperlinearity of the palms and soles. At 4 years of age minimal signs of ichthyosis
Noguera-Morel L et al. [60], 2016, Spain	c.1303C>T p.(His435Tyr) (homozygous missense mutation)	M	Term	At birth	Collodion baby	n.r.	At 5 years of age, minimal facial erythema and hyperlinearity of the palms and soles
Zao L et al. [62], 2022, China	c.844C>T p.(R282W) c.1189C>T p.(R397C) (compound heterozygous)	M	Term	At birth	Collodion baby associated with bilateral ectropion	n.r.	At 2 months of age residual plate-like scales on the scalp, mild erythema with fine scaling, especially on the back, axillary, and knees and hyperlinearity of the palms and soles
Tang et al. [54], 2021, China	c.1084C>T in exon 10th c.1137-2delA around exon 11th (compound heterozygous)	F	Term	At birth	Collodion baby	n.r.	At 3 years of age generalized LI with brown, plate-like. Hyperlinearity of palms and soles.
Fioretti T et al. [64], 2020, Italy	c.76_85del p.(Thr26Serfs) delExon3 (compound heterozygous)	F	n.r.	At birth	Collodion baby	Hydrating and keratolytic agents	In infancy, large white-gray scaling on the whole body, with areas of erythema and significant hyperkeratosis persistent in the adult age.
Takeichi T et al. [65], 2022, Japan	c.235G>T p.(Glu79*)/c.1189C>T p.(Arg397Cys) (compound heterozygous)	M	Term	At birth	Collodion membrane over his entire body surface, with moderate fissuring at the joints. During the first 10 days of life, the thick scales gradually desquamated	n.r.	At 2 years of age, extremely mild generalized ichthyosis and overlying mild fine scaling
Takeichi T et al. [65], 2022, Japan	c.1295A>G p.(Tyr432- Cys) c.1138delG p.(Asp380Thrfs*3) (compound heterozygous)	M	Term	At 12 months of age	Lamellar ichthyosis	Emollients	At 11 years of age, very mild fine scales on the trunk and extremities
Bučková H et al. [66], 2016, Czech Republic	c.844C>TR	M	Term	At birth	Collodion baby	n.r.	At 14 years of age, brown plate-like scales. Palmoplantar hyperkeratosis
Patient 2 (2025)	truncating variant: c.1084C>T p.(Arg362Ter) a in-frame deletion: c.543_545del p.(Ile181del) (compound heterozygous)	F	Term	At birth	Skin dehydration with a shiny, translucent membrane covering nearly the entire body surface, thick whitish desquamating plaques over the body, mild ectropion and eclabium	Emollient creams every 4–6 h, along with lubricating eye drops for ectropion management	At age 4 months: adequate growth; minimal areas of erythema and dryness on the upper eyelids.

F: female; M: male; n.r.: not reported.

## Data Availability

The datasets and materials generated and/or analysed during the current study are available from the corresponding author upon reasonable request.

## Supplementary Materials

The following supporting information can be downloaded at https://www.mdpi.com/article/10.3390/jcm15145556/s1: File S1: Bioinformatic Analysis of Identified Variants in *TGM1*; File S2: Bioinformatic Analysis of Identified Variants in *CYP4F22* [25,26,32].

## Abbreviations

The following abbreviations are used in this manuscript:

ARCI	Autosomal Recessive Congenital Ichthyosis
CI	Congenital Ichthyosis
CIE	Congenital Ichthyosiform Erythroderma
gnomAD	Genome Aggregation Database
HI	Harlequin Ichthyosis
IV	Ichthyosis Vulgaris
iv	Intravenous
KPI	Keratinopathic Ichtyoses
LI	Lamellar Ichthyosis
NGS	Next-Generation Sequencing

## References

[1] Akiyama M., Choate K., Hernández-Martín Á., Aldwin-Easton M., Bodemer C., Gostyński A., Hovnanian A., Ishida-Yamamoto A., Malovitski K., O’Toole E.A. (2025). Nonsyndromic epidermal differentiation disorders: A new classification toward pathogenesis-based therapy. Br. J. Dermatol..

[2] Oji V., Tadini G., Akiyama M., Blanchet Bardon C., Bodemer C., Bourrat E., Coudiere P., DiGiovanna J.J., Elias P., Fischer J. (2010). Revised nomenclature and classification of inherited ichthyoses: Results of the First Ichthyosis Consensus Conference in Sorèze 2009. J. Am. Acad. Dermatol..

[3] Takeichi T., Akiyama M. (2016). Inherited ichthyosis: Non-syndromic forms. J. Dermatol..

[4] Yoneda K. (2016). Inherited ichthyosis: Syndromic forms. J. Dermatol..

[5] Israeli S., Goldberg I., Fuchs-Telem D., Bergman R., Indelman M., Bitterman-Deutsch O., Harel A., Mashiach Y., Sarig O., Sprecher E. (2013). Non-syndromic autosomal recessive congenital ichthyosis in the Israeli population. Clin. Exp. Dermatol..

[6] Fischer J., Bourrat E. (2020). Genetics of Inherited Ichthyoses and Related Diseases. Acta Derm. Venereol..

[7] Valutazione Antropometrica Neonatale. Riferimento Carte INeS. http://www.inescharts.com.

[8] World Health Organization (2021). Child Growth Standards. https://www.who.int/tools/child-growth-standards/standards.

[9] Perez G., Barber G.P., Benet-Pages A., Casper J., Clawson H., Diekhans M., Fischer C., Gonzalez J.N., Hinrichs A.S., Lee C.M. (2025). The UCSC Genome Browser database: 2025 update. Nucleic Acids Res..

[10] Human Gene Mutation Database (HGMD). http://www.hgmd.org.

[11] Rehder C., Bean L.J.H., Bick D., Chao E., Chung W., Das S., O’Daniel J., Rehm H., Shashi V., Vincent L.M. (2021). Next-generation sequencing for constitutional variants in the clinical laboratory, 2021 revision: A technical standard of the American College of Medical Genetics and Genomics (ACMG). Genet. Med..

[12] Firth H.V. (2009). DECIPHER: Database of Chromosomal Imbalance and Phenotype in Humans using Ensembl Resources. Am. J. Hum. Genet..

[13] ClinVar. https://www.ncbi.nlm.nih.gov/clinvar/.

[14] Stenson P.D., Mort M., Ball E.V., Evans K., Hayden M., Heywood S., Hussain M., Phillips A.D., Cooper D.N. (2017). The Human Gene Mutation Database: Towards a comprehensive repository of inherited mutation data for medical research, genetic diagnosis and next-generation sequencing studies. Hum. Genet..

[15] LOVD. https://www.lovd.nl/.

[16] Troina Database. https://gvarianti.oasi.en.it/genome_browser.php.

[17] Genome Aggregation Database (gnomAD), Version 4.1. Broad Institute. https://gnomad.broadinstitute.org/.

[18] (2015). Quality Management Systems.

[19] Yates A., Akanni W., Amode M.R., Barrell D., Billis K., Carvalho-Silva D., Cummins C., Clapham P., Fitzgerald S., Gil L. (2016). Ensembl 2016. Nucleic Acids Res..

[20] Ng P.C., Henikoff S. (2001). Predicting deleterious amino acid substitutions. Genome Res..

[21] Adzhubei I.A., Schmidt S., Peshkin L., Ramensky V.E., Gerasimova A., Bork P., Kondrashov A.S., Sunyaev S.R. (2010). A method and server for predicting damaging missense mutations. Nat. Methods.

[22] Kircher M., Witten D.M., Jain P., O’Roak B.J., Cooper G.M., Shendure J. (2014). A general framework for estimating the relative pathogenicity of human genetic variants. Nat. Genet..

[23] Ioannidis N.M., Rothstein J.H., Pejaver V., Middha S., McDonnell S.K., Baheti S., Musolf A., Li Q., Holzinger E., Karyadi D. (2016). REVEL: An Ensemble Method for Predicting the Pathogenicity of Rare Missense Variants. Am. J. Hum. Genet..

[24] Dong C., Wei P., Jian X., Gibbs R., Boerwinkle E., Wang K., Liu X. (2015). Comparison and integration of deleteriousness prediction methods for nonsynonymous SNVs in whole exome sequencing studies. Hum. Mol. Genet..

[25] Reva B., Antipin Y., Sander C. (2011). Predicting the functional impact of protein mutations: Application to cancer genomics. Nucleic Acids Res..

[26] ClinVar (2025). NM_000359.3(TGM1):c.1147G>A (p.Val383Met).

[27] Kopanos C., Tsiolkas V., Kouris A., Chapple C.E., Aguilera M.A., Meyer R., Massouras A. (2019). VarSome: The human genomic variant search engine. Bioinformatics.

[28] Dyer S.C., Austine-Orimoloye O., Azov A.G., Barba M., Barnes I., Barrera-Enriquez V.P., Becker A., Bennett R., Beracochea M., Berry A. (2025). Ensembl 2025. Nucleic Acids Res..

[29] Terrinoni A., Serra V., Codispoti A., Talamonti E., Bui L., Palombo R., Sette M., Campione E., Didona B., Annicchiarico-Petruzzelli M. (2012). Novel transglutaminase 1 mutations in patients affected by lamellar ichthyosis. Cell Death Dis..

[30] Ebrahimi Samani S., Tatsukawa H., Hitomi K., Kaartinen M.T. (2024). Transglutaminase 1: Emerging Functions beyond Skin. Int. J. Mol. Sci..

[31] Petit E., Huber M., Rochat A., Bodemer C., Teillac-Hamel D., Müh J.P., Revuz J., Barrandon Y., Lathrop M., de Prost Y. (1997). Three novel point mutations in the keratinocyte transglutaminase (TGK) gene in lamellar ichthyosis: Significance for mutant transcript level, TGK immunodetection and activity. Eur. J. Hum. Genet..

[32] Alallasi S.R., Kokandi A.A., Banagnapali B., Shaik N.A., Al-Shehri B.A., Alrayes N.M., Al-Aama J.Y., Jelani M. (2019). Exome Analysis Identifies a Novel Compound Heterozygous Alteration in TGM1 Gene Leading to Lamellar Ichthyosis in a Child from Saudi Arabia: Case Presentation. Front. Pediatr..

[33] Richards S., Aziz N., Bale S., Bick D., Das S., Gastier-Foster J., Grody W.W., Hegde M., Lyon E., Spector E. (2015). Standards and guidelines for the interpretation of sequence variants: A joint consensus recommendation of the American College of Medical Genetics and Genomics and the Association for Molecular Pathology. Genet. Med..

[34] Hackett B.C., Fitzgerald D., Watson R.M., Hol F.A., Irvine A.D. (2010). Genotype-phenotype correlations with TGM1: Clustering of mutations in the bathing suit ichthyosis and self-healing collodion baby variants of lamellar ichthyosis. Br. J. Dermatol..

[35] Sugiura K., Suga Y., Akiyama M. (2013). Very mild lamellar ichthyosis with compound heterozygous TGM1 mutations including the novel missense mutation p.Leu693Phe. J. Dermatol. Sci..

[36] Shevchenko Y.O., Compton J.G., Toro J.R., DiGiovanna J.J., Bale S.J. (2000). Splice-site mutation in TGM1 in congenital recessive ichthyosis in American families: Molecular, genetic, genealogic, and clinical studies. Hum. Genet..

[37] Cserhalmi-Friedman P.B., Milstone L.M., Christiano A.M. (2001). Diagnosis of autosomal recessive lamellar ichthyosis with mutations in the TGM1 gene. Br. J. Dermatol..

[38] Shawky R.M., Sayed N.S., Elhawary N.A. (2004). Mutations in transglutaminase 1 gene in autosomal recessive congenital ichthyosis in Egyptian families. Dis. Markers.

[39] Mazereeuw-Hautier J., Aufenvenne K., Deraison C., Ahvazi B., Oji V., Traupe H., Hovnanian A. (2009). Acral self-healing collodion baby: Report of a new clinical phenotype caused by a novel TGM1 mutation. Br. J. Dermatol..

[40] Esposito G., De Falco F., Brazzelli V., Montanari L., Larizza D., Salvatore F. (2009). Autosomal recessive congenital ichthyosis and congenital hypothyroidism in a Tunisian patient with a nonsense mutation in TGM1. J. Dermatol. Sci..

[41] Cao X., Lin Z., Yang H., Bu D., Tu P., Chen L., Wu H., Yang Y. (2009). New mutations in the transglutaminase 1 gene in three families with lamellar ichthyosis. Clin. Exp. Dermatol..

[42] Rodríguez-Pazos L., Ginarte M., Vega-Gliemmo A., Toribio J. (2009). Lamellar ichthyosis with a novel homozygous C-terminal mutation in the transglutaminase-1 gene. Int. J. Dermatol..

[43] Fachal L., Rodríguez-Pazos L., Ginarte M., Beiras A., Suárez-Peñaranda J.M., Toribio J., Carracedo Á., Vega A. (2012). Characterization of TGM1 c.984+1G>A mutation identified in a homozygous carrier of lamellar ichthyosis. Int. J. Dermatol..

[44] Al-Naamani A., Al-Waily A., Al-Kindi M., Al-Awadi M., Al-Yahyaee S.A. (2013). Transglutaminase-1 mutations in Omani families with lamellar ichthyosis. Med. Princ. Pract..

[45] Park S.H., Shin J.Y., Park Y.M., Youn Y.A., Kim M. (2013). A novel TGM1 splicing mutation in a collodion baby with cicatricial ectropion. Can. J. Ophthalmol..

[46] Benmously-Mlika R., Zaouak A., Mrad R., Laaroussi N., Abdelhak S., Hovnanian A., Mokhtar I. (2014). Bathing suit ichthyosis caused by a TGM1 mutation in a Tunisian child. Int. J. Dermatol..

[47] Tanahashi K., Sugiura K., Asagoe K., Aoyama Y., Iwatsuki K., Akiyama M. (2014). Novel TGM1 missense mutation p.Arg727Gln in a case of self-healing collodion baby. Acta Derm. Venereol..

[48] Suga Y., Tsuda T., Nagai M., Sakaguchi Y., Jitsukawa O., Yamamoto M., Hitomi K., Yamanishi K. (2015). Lamellar ichthyosis with pseudoexon activation in the transglutaminase 1 gene. J. Dermatol..

[49] Bastaki F., Mohamed M., Nair P., Saif F., Mustafa E.M., Bizzari S., Al-Ali M.T., Hamzeh A.R. (2017). Summary of mutations underlying autosomal recessive congenital ichthyoses (ARCI) in Arabs with four novel mutations in ARCI-related genes from the United Arab Emirates. Int. J. Dermatol..

[50] Zaouak A., Abdessalem G., Mkaouar R., Messaoud O., Abdelhak S., Hammami H., Fenniche S. (2019). Congenital lamellar ichthyosis in Tunisia associated with vitamin D rickets caused by a founder nonsense mutation in the TGM1 gene. Int. J. Dermatol..

[51] Diep Q.M., Luong L.H., Tran T.H., Dinh O.T.L., Nguyen H.Q., Bui T.H., Ta T.V., Tran V.K. (2020). A case of self-improving collodion ichthyosis in Vietnam. Pediatr. Dermatol..

[52] Guo Y., Xiao Z., Hu X., Liu Y., Chen G. (2025). A case report and literature review of self-improving collodion baby in the newborn. Medicine.

[53] Diociaiuti A., Corbeddu M., Rossi S., Pisaneschi E., Cesario C., Condorelli A.G., Samela T., Giancristoforo S., Angioni A., Zambruno G. (2024). Cross-Sectional Study on Autosomal Recessive Congenital Ichthyoses: Association of Genotype with Disease Severity, Phenotypic, and Ultrastructural Features in 74 Italian Patients. Dermatology.

[54] Tang H., Shi X., Zhang G. (2021). Novel compound heterozygous mutations in the CYP4F22 gene in a patient with autosomal recessive congenital ichthyosis. Clin. Case Rep..

[55] Mutation Update for CYP4F22 Variants Associated with Autosomal Recessive Congenital Ichthyosis—PubMed [Internet]. https://pubmed.ncbi.nlm.nih.gov/30011118/.

[56] Fluhr J.W., Moore D.J., Lane M.E., Lachmann N., Rawlings A.V. (2024). Epidermal barrier function in dry, flaky and sensitive skin: A narrative review. J. Eur. Acad. Dermatol. Venereol..

[57] Liu Z., Qin X., Wang X., Zhang J., Yang B. (2025). Mechanisms and repair of skin barrier dysfunction: The TLC strategy. Int. J. Dermatol..

[58] Dumenigo A., Rusk A., Marathe K. (2022). CYP4F22-Related Autosomal Recessive Congenital Ichthyosis: Clinical Presentation. Cureus.

[59] Swink S.M., Hurley M., Haynes D., Larijani M. (2024). Autosomal recessive congenital ichthyosis due to novel CYP4F22 mutation presenting with a collodion membrane and ocular manifestations. Pediatr. Dermatol..

[60] Noguera-Morel L., Feito-Rodríguez M., Maldonado-Cid P., García-Miñáur S., Kamsteeg E.J., González-Sarmiento R., De Lucas-Laguna R., Hernández-Martín A., Torrelo A. (2016). Two Cases of Autosomal Recessive Congenital Ichthyosis due to CYP4F22 Mutations: Expanding the Genotype of Self-Healing Collodion Baby. Pediatr. Dermatol..

[61] Gruber R., Rainer G., Weiss A., Udvardi A., Thiele H., Eckl K.M., Schupart R., Nürnberg P., Zschocke J., Schmuth M. (2017). Morphological alterations in two siblings with autosomal recessive congenital ichthyosis associated with CYP4F22 mutations. Br. J. Dermatol..

[62] Zhao L., Wang C., Zhang Y., Li J., Liu H., Feng D. (2022). Whole-exome sequencing identified a novel pathogenic mutation of the CYP4F22 gene in a Chinese patient with autosomal recessive congenital ichthyosis and in vitro study of the mutant CYP4F22 protein. J. Dermatol..

[63] Feng C., Wang H., Lee M., Zhao J., Lin Z., Yang Y. (2017). Two missense mutations in CYP4F22 in autosomal recessive congenital ichthyosis. Clin. Exp. Dermatol..

[64] Fioretti T., Auricchio L., Piccirillo A., Vitiello G., Ambrosio A., Cattaneo F., Ammendola R., Esposito G. (2020). Multi-Gene Next-Generation Sequencing for Molecular Diagnosis of Autosomal Recessive Congenital Ichthyosis: A Genotype-Phenotype Study of Four Italian Patients. Diagnostics.

[65] Takeichi T., Ohno Y., Tanahashi K., Ito Y., Shiraishi K., Utsunomiya R., Yoshida S., Ikeda K., Nomura H., Morizane S. (2022). Ceramide Analysis in Combination with Genetic Testing May Provide a Precise Diagnosis for Self-Healing Collodion Babies. J. Lipid Res..

[66] Bučková H., Nosková H., Borská R., Réblová K., Pinková B., Zapletalová E., Kopečková L., Horký O., Němečková J., Gaillyová R. (2016). Autosomal recessive congenital ichthyoses in the Czech Republic. Br. J. Dermatol..

[67] Liu Z., Zhu L., Roberts R., Tong W. (2019). Toward Clinical Implementation of Next-Generation Sequencing-Based Genetic Testing in Rare Diseases: Where Are We?. Trends Genet..

[68] Serra G., Memo L., Cavicchioli P., Cutrone M., Giuffrè M., La Torre M.L., Schierz I.A., Corsello G. (2022). Novel mutations of the ABCA12, KRT1 and ST14 genes in three unrelated newborns showing congenital ichthyosis. Ital. J. Pediatr..

[69] Schierz I.A.M., Giuffrè M., Cimador M., D’Alessandro M.M., Serra G., Favata F., Antona V., Piro E., Corsello G. (2022). Hypertrophic pyloric stenosis masked by kidney failure in a male infant with a contiguous gene deletion syndrome at Xp22.31 involving the steroid sulfatase gene: Case report. Ital. J. Pediatr..

[70] Serra G., Antona V., D’Alessandro M.M., Maggio M.C., Verde V., Corsello G. (2021). Novel SCNN1A gene splicing-site mutation causing autosomal recessive pseudohypoaldosteronism type 1 (PHA1) in two Italian patients belonging to the same small town. Ital. J. Pediatr..

[71] Diociaiuti A., El Hachem M., Pisaneschi E., Giancristoforo S., Genovese S., Sirleto P., Boldrini R., Angioni A. (2016). Role of molecular testing in the multidisciplinary diagnostic approach of ichthyosis. Orphanet J. Rare Dis..

[72] Vahlquist A., Bygum A., Gånemo A., Virtanen M., Hellström-Pigg M., Strauss G., Brandrup F., Fischer J. (2010). Genotypic and clinical spectrum of self-improving collodion ichthyosis: ALOX12B, ALOXE3, and TGM1 mutations in Scandinavian patients. J. Investig. Dermatol..

[73] Pensabene M., Di Pace M.R., Baldanza F., Grasso F., Patti M., Sergio M., La Placa S., Giuffre’ M., Serra G., Casuccio A. (2022). Quality of life improving after propranolol treatment in patients with Infantile Hemangiomas. Ital. J. Pediatr..

