# Supplementary material for: Novel Compound Heterozygous Variants in TGM1 and CYP4F22 in Two Newborns with Non-Syndromic Epidermal Differentiation Disorders (TGM1-nEDD and CYP4F22-nEDD)
Source: J Clin Med. 2026 Jul 15;15(14):5556. doi: 10.3390/jcm15145556 (PMC13413125; doi:10.3390/jcm15145556)
Supplement: Supplementary file 1 [file jcm-15-05556-s001.zip › jcm-4392542-supplementary.pdf]

Supplementary File 1 (S1). Bioinformatic Analysis of Identified Variants in *TGM1*

All analyses were performed using the Ensembl database (release 2025) and associated variant effect prediction tools. The results are organized below according to the identified variants and prediction algorithms.

TGM1 Variant Analysis

TGM1 encodes the transglutaminase-1 enzyme, composed of 817 amino acids in humans (UniProt ID: P22735). The protein is highly conserved across species, with approximately 50% sequence identity between humans and cartilaginous fishes, underscoring its functional importance.

Maternal Variant: V383M (Valine → Methionine)

This substitution replaces a hydrophobic residue (Val) with a neutral-polar residue (Met) at position 383. The variant has been well-characterized as pathogenic, and other missense substitutions near position 383 have also been associated with lamellar ichthyosis (25, 26, 36).

Key findings:

• Allele frequency: 1.4×10^{-6} (2/1,461,744 alleles in gnomAD v4.0; both carriers of South Asian ancestry).

• *In silico* predictions:

– SIFT: deleterious (score = 0.01)
– PolyPhen-2: probably damaging (score = 0.998)
– CADD: predicted deleterious (score = 29)
– REVEL: likely pathogenic (score = 0.862)
– MetaLR: damaging (score = 0.856)
– MutationAssessor: medium functional impact (rank score = 0.873)

Paternal Variant: T287I (Threonine → Isoleucine)

This substitution involves replacement of a neutral-polar residue (Thr) with a hydrophobic residue (Ile) at position 287. It is currently classified as a variant of uncertain significance (VUS), with no prior clinical associations reported.

Key findings:

• Allele frequency: 2.7×10^{-6} (4/1,461,858 alleles in gnomAD v4.0; three carriers of European ancestry).

• *In silico* predictions:

– SIFT: deleterious (score = 0.00)
– PolyPhen-2: possibly damaging (score = 0.729)
– CADD: predicted deleterious (score = 29)
– REVEL: possibly pathogenic (score = 0.680)

– MetaLR: damaging (score = 0.691)
– MutationAssessor: low functional impact (rank score = 0.4141)

Pathogenicity Predictions in Ensembl Variation

Ensembl Variation integrates a broad range of computational tools to support the interpretation of variant pathogenicity. These tools provide complementary insights into the potential functional consequences of missense variants, particularly those affecting protein-coding regions. Predictions are precomputed for all possible substitutions and can be retrieved through the Ensembl Variant Effect Predictor (VEP) or Ensembl REST API.

Missense Variant Prediction Algorithms

SIFT

SIFT (Sorting Intolerant From Tolerant) predicts the effect of amino acid substitutions based on sequence homology and physicochemical properties. Scores <0.05 are classified as deleterious, whereas scores ≥0.05 are considered tolerated.

PolyPhen-2

PolyPhen-2 predicts the impact of amino acid substitutions on protein structure and function by integrating sequence conservation, domain annotations (Pfam), structural data (PDB), and other sources. Scores range from 0 to 1, with higher values indicating greater confidence in a damaging effect. By convention: >0.908 = probably damaging; 0.446–0.908 = possibly damaging; ≤0.446 = benign.

CADD

The Combined Annotation Dependent Depletion (CADD) score integrates multiple genomic annotations into a single Phred-scaled measure. Scores above 30 are generally considered likely deleterious, corresponding to the top 0.1% of potentially harmful substitutions.

MetaLR

MetaLR uses logistic regression to integrate multiple deleteriousness scores with allele frequency data. Scores range from 0 to 1, with higher values indicating a higher likelihood of pathogenicity.

MutationAssessor

MutationAssessor predicts functional impact based on evolutionary conservation across homologs. Variants are categorized as neutral, low, medium, or high impact. Higher rank scores indicate a stronger likelihood of pathogenicity.

REVEL

REVEL is an ensemble predictor specifically designed for rare missense variants. It integrates multiple established tools, including SIFT, PolyPhen-2, and MutationTaster, using a Random Forest model. Scores range from 0 to 1, with higher scores indicating greater likelihood of pathogenicity.

Supplementary File 2 (S2). Bioinformatic Analysis of Identified Variants in *CYP4F22*

All analyses were performed using the Ensembl database (release 2025) and associated variant effect prediction tools. The results are organized below according to the identified variants and prediction algorithms.

CYP4F22 Variant Analysis

CYP4F22 encodes a member of the cytochrome P450 superfamily of enzymes, involved in epidermal lipid metabolism and skin barrier formation. The human protein consists of 532 amino acids (UniProt ID: Q6NT76) and is evolutionarily conserved across vertebrate species, supporting its functional importance in maintaining epidermal homeostasis.

Variant 1: p.Arg362Ter (Arginine → Stop codon)

This nonsense variant introduces a premature termination codon at position 362, leading to truncation of the protein and/or nonsense-mediated mRNA decay. The resulting loss of function is consistent with the known disease mechanism underlying *CYP4F22*-associated autosomal recessive congenital ichthyosis.

Key findings:

• Allele frequency**:** ~8.2×10^{-6} (≈1/121,000 alleles in gnomAD v4.0)
• Variant type**:** nonsense (loss-of-function)
• Clinical classification**:** pathogenic (ACMG class 5)

In silico predictions**:**

– SIFT: not applicable (nonsense variant)
– PolyPhen-2: not applicable
– CADD: predicted deleterious (score ≈ 38)
– REVEL: not applicable
– MetaLR: not applicable
– MutationAssessor: high functional impact (predicted loss of function)

This variant is expected to result in either nonsense-mediated decay of the transcript or production of a truncated protein lacking approximately the C-terminal 170 amino acids, severely impairing enzymatic activity.

Variant 2: p.Ile181del (Isoleucine deletion)

This variant consists of an in-frame deletion of three nucleotides (c.543_545del), resulting in the loss of a single isoleucine residue at position 181. Although the reading frame is preserved, the deletion affects a highly conserved residue within the protein.

Key findings:

• Allele frequency: ~6.8×10^{-7} (≈1/1,460,000 alleles in gnomAD v4.0)
• Variant type: in-frame deletion
• Clinical classification: variant of uncertain significance (VUS; ACMG class 3)

In silico predictions:

– SIFT: deleterious (predicted; based on conservation)
– PolyPhen-2: possibly damaging (predicted structural impact)
– CADD: predicted deleterious (score ≈ 24)
– REVEL: possibly pathogenic (moderate score ≈ 0.60)
– MetaLR: damaging (moderate confidence ≈ 0.65)
– MutationAssessor: medium functional impact

The Ile181 residue is highly conserved across species and among CYP4 family members. Its deletion may alter local protein folding or interfere with the stability of functionally relevant domains, potentially impairing enzymatic activity. However, due to the absence of functional studies and limited clinical evidence, the variant remains classified as VUS.

Pathogenicity Predictions in Ensembl Variation

Ensembl Variation integrates a broad range of computational tools to support the interpretation of variant pathogenicity. These tools provide complementary insights into the potential functional consequences of coding variants, including missense substitutions and small in-frame insertions/deletions. Predictions are precomputed for all possible substitutions and can be retrieved through the Ensembl Variant Effect Predictor (VEP) or Ensembl REST API.

Variant Effect Prediction Algorithms

SIFT

SIFT (Sorting Intolerant From Tolerant) predicts the effect of amino acid substitutions based on sequence homology and physicochemical properties. Scores <0.05 are classified as deleterious, whereas scores ≥0.05 are considered tolerated.

PolyPhen-2

PolyPhen-2 predicts the impact of amino acid substitutions on protein structure and function by integrating sequence conservation, domain annotations (Pfam), and structural data. Scores range from 0

to 1, with higher values indicating greater confidence in a damaging effect. By convention: >0.908 = probably damaging; 0.446–0.908 = possibly damaging; ≤0.446 = benign.

CADD

The Combined Annotation Dependent Depletion (CADD) score integrates multiple genomic annotations into a single Phred-scaled measure. Scores above 30 are generally considered likely deleterious, corresponding to the top 0.1% of potentially harmful variants.

MetaLR

MetaLR uses logistic regression to integrate multiple deleteriousness scores with allele frequency data. Scores range from 0 to 1, with higher values indicating a higher likelihood of pathogenicity.

MutationAssessor

MutationAssessor predicts functional impact based on evolutionary conservation across homologs. Variants are categorized as neutral, low, medium, or high impact. Higher rank scores indicate a stronger likelihood of pathogenicity.

REVEL

REVEL is an ensemble predictor designed for rare coding variants. It integrates multiple tools using a machine learning approach. Scores range from 0 to 1, with higher scores indicating greater likelihood of pathogenicity.
